# Interaction of hepatic stellate cells with neutrophils and macrophages in the liver following oncogenic *kras* activation in transgenic zebrafish

**DOI:** 10.1038/s41598-018-26612-0

**Published:** 2018-05-31

**Authors:** Qiqi Yang, Chuan Yan, Zhiyuan Gong

**Affiliations:** 1 Department of Biological Sciences, National University of Singapore, Singapore, Singapore; 2 National University of Singapore graduate school for integrative sciences and engineering, National University of Singapore, Singapore, Singapore

## Abstract

Activation of hepatic stellate cells (HSC) plays a crucial role in the liver disease progression from liver fibrosis/cirrhosis to cancer. Here, we found a rapid change of microenvironment after *kras*^*V12*^-induction in zebrafish liver with progressively increased stromal cell number and enlarged liver size. Neutrophils and macrophages exhibited a faster response than HSCs. By manipulating the numbers of neutrophils and macrophages through morpholino knockdown, we found that macrophages contributed to both HSC survival and activation while neutrophils appear to be only required for HSC activation. Serotonin, which is essential for HSC survival and activation, was found up-regulated in hepatocytes and macrophages, but not in neutrophils after *kras*^*V12*^ induction. Serotonin receptor was highly expressed in HSCs; increase of the receptor activity by an agonist stimulated HSCs and oncogenic growth of the liver while an opposite effect was observed with an antagonist. Activated HSCs promoted the pro-tumorigenesis functions of neutrophils and macrophages through secretion of Tgfb1. Overall, these observations elucidated a cellular interaction in microenvironment where that upregulated serotonin in hepatocytes and macrophages activated HSCs. Since the microenvironment crosstalk plays a vital role in manipulation of liver carcinogenesis, the underlying mechanism may provide potential therapeutic targets for liver diseases.

## Introduction

Tumor microenvironment is important in tumor initiation and progression. Hepatic stellate cells (HSCs), liver-specific mesenchymal cells, are an important component of tumor microenvironment and the stromal dynamics is also a key determinant of liver cancer progression^1^. In normal liver, HSCs are quiescent; upon liver injury, HSCs can be activated by various cytokines, such as TGFB (transforming growth factor b), IL1B (interleukin 1b) and TNF (tumor necrosis factor)^2^. HSC activation results in increasing extracellular matrix remodeling capabilities, as observed in non-alcoholic steatohepatitis patients^3^. There is also a strong relationship between activated HSCs and oncogenic hepatocytes. HSCs are the pre-eminent matrix-producing cells in liver fibrosis, potently secreting TGFB1 and PDGF, thus mediating liver fibrosis as well as hepatocyte proliferation^4^.

In hepatocellular carcinoma (HCC), activated HSCs induce phenotypic changes in oncogenic hepatocytes, notably through the production of growth factors and cytokines such as HGF (hepatocyte growth factor) and IL6 in favor of tumor cell proliferation^5^. Non-neoplastic stromal composition serves as a reliable prognostic predictor in HCC patient survival and recurrence^6,7^. Densities of intratumoral endothelial cells, tumor associated macrophages (TAMs), tumor associated neutrophils (TANs), CD8+ T-cells and activated HSCs are significantly higher in patients with liver diseases and they are also indicators of poor disease prognosis^8–11^. Curiously, various components of the tumor microenvironment are capable of influencing one another to build a complex signaling network. For example, TAMs and TANs are simultaneously activated to a pro-tumor gene expression profile during carcinogenesis and could affect each other^12^. Interestingly, both TAMs and TANs have been demonstrated to be able to influence HSCs in a non-neoplastic context. TAMs activate NFkB signaling in HSCs and thereby promote liver fibrosis via HSC-mediated extracellular matrix remodeling^13^. TAN-derived superoxide anion induces lipid peroxidation and stimulates collagen synthesis in HSCs^14^.

The zebrafish has been increasingly recognized as an alternative animal model in cancer studies^15^. Previously our laboratory has generated several inducible HCC models by transgenic expression of an oncogene in hepatocytes in zebrafish^16–19^. These zebraifsh models showed close histological resemblance to human HCC and share conserved molecular signatures with advanced human HCC^20,21^. Moreover, the inducible nature of these transgenic HCC model allows the oncogene to be switched on at a desired timing, providing an excellent model to study HCC initiation events. In addition, the transparent nature of zebrafish larvae and the availability of various transgenic reporter zebrafish with expression of a fluorescence protein marker in specific cell lineage such as HSCs, neutrophils or macrophages provide excellent tools to elucidate interactions of different microenvironment components in a neoplastic context^22–24^. In this study, we attempt to elucidate the microenvironment interaction among hepatocytes, HSCs, TAMs and TANs during early stages of liver tumorigenesis. By using several transgenic zebrafish lines, we found that along with *kras*^*V12*^-expressing oncogenic hepatocytes (OHs), macrophages could activate HSCs through serotonin-mediated signaling. Interestingly, HSC activation resulted in up-regulation of Tgfb1 expression, which in turn maintained pro-tumor gene expression in TANs and TAMs and thereby promoted carcinogenesis.

## Results

### Rapid modification of microenvironment following *kras*^*V12*^-induced liver tumorigenesis

**Figure 1:**
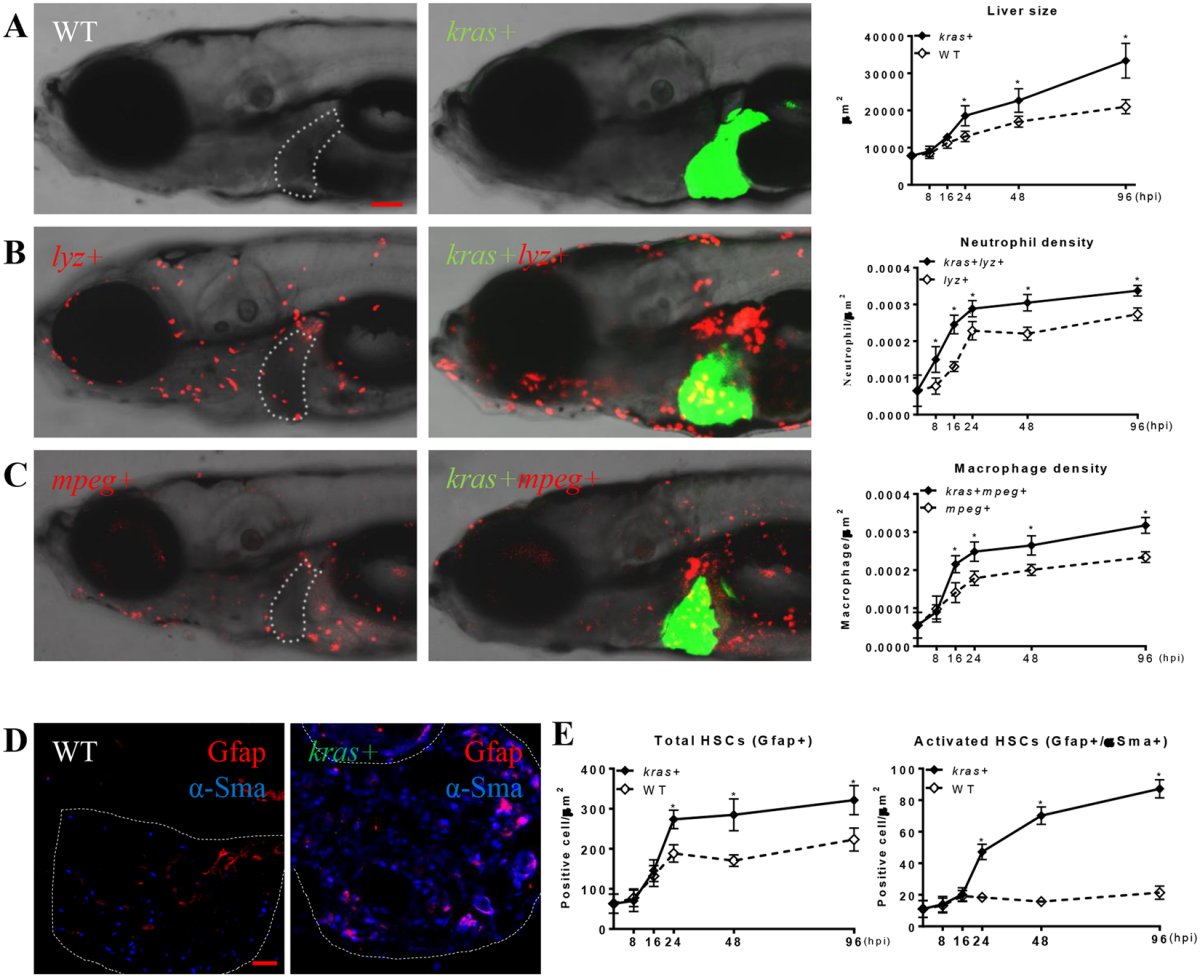
Rapid modification of tumor microenvironment following oncogenic *kras*^*V12*^ expression. *Kras*+ fish were crossed with *lyz*+ or *mpeg*+ fish. Larvae from the crosses were treated with 20 μg/ml dox from 3 dpf and fixed at 8, 16, 24, 48, 96 hpi. At each time point, 10 fish/group were mounted in methylcellulose and imaged at left lateral side under a confocal microscope. Neutrophils, macrophages and HSCs were counted manually based on DsRed/mCherry fluorescence and Gfap or α-Sma staining. (**A**) Representative images of the WT (left) or *kras*+ (middle) larvae at 96 hpi and quantification of 2D liver size (right) from 8 hpi to 96 hpi, (**B**) Representative images of *lyz*+ larvae in the WT (left) or *kras*+ (middle) background at 96 hpi and quantification of neutrophil density in the liver from 8 hpi to 96 hpi. (**C**) Representative images of *mpeg*+ larvae in the WT (left) or *kras*+ (middle) background at 96 hpi and quantification of macrophage density in the liver from 8 hpi to 96 hpi. (**D**) Representative images of IF co-staining of GFAP (red) and α-Sma (blue) in liver sections of zebrafish larvae at 96 hpi. (**E**) Quantification of total HSC number (Gfap+) and activated HSCs (Gfap+ and α-Sma+) in liver sections in larvae from 8 hpi to 96 hpi. Livers are outlined when GFP signal is not available. In all experiments, n = 20 for each group. *P < 0.05. Error bars represent biological replicates. Scale bars: 20 μm.

In human HCC patients, there are high densities of TANs, TAMs and HSCs in the liver tumors and these correlate to poor prognosis^8–11^. To investigate if *kras*^*V12*^-intiated liver tumorigenesis would induce similar microenvironment modification in our transgenic zebrafish model, infiltration of neutrophils and macrophages as well as HSC density and activation were examined using different reporter transgenic lines as described in Methods. By crossing either *lyz*+ or *mpeg*+ transgenic reporter line with the *kras*+ zebrafish, infiltration of neutrophils and macrophages into *kras*^*V12*^-expressing livers was visualized at different time. Consistent with our previous study^25^, neutrophil infiltration was significantly increased from 8 hours post-induction (hpi), preceding the onset of *kras*^*V12*^-induced liver enlargement at 24 hpi (Fig. 1A,B). Similarly, macrophage infiltration became significant from 16 hpi (Fig. 1C). To examine HSC density and activation, a combination of HSC marker proteins, glial fibrillary acidic protein (Gfap) and α-smooth muscle actin (α-Sma), was used to identify HSCs in *kras*^*V12*^-expressing livers (Fig. 1D). Gfap+ cells in the liver demarcate both quiescent and activated HSCs^22^, while α-Sma marks only activated HSC^26^. Supplementary Figure S1A shows co-localization of hepatocyte-specific gfp, DAPI labeled nuclei and α-Sma. It is apparent that α-Sma is located in both cytoplasm and nuclei and this is consistent with an earlier observation in human mesenchymal stromal cells^27^. It is also interesting to note that α-Sma is also expressed at a low level in the liver (mostly in hepatocytes) (Fig. 1D). This is consistent with an earlier report in a mouse liver tumor model^28^. Quantification of Gfap and α-Sma staining is shown in Fig. 1E and it appeared that both total HSC density and the ratio of activated HSCs were increased significantly in *kras*^*V12*^-expressing livers, compared to wildtype livers at 24 hpi. In our previous study, we demonstrated that *kras*+ fish showed early HCC phenotype at 7 dpf and 96 hpi^25^. Hence, *kras*^*V12*^-expressing induced rapid microenvironment modifications, as indicated by sequential occurrence of increased infiltration of neutrophils and macrophages, followed by proliferation and activation of HSCs.

### Regulation of HSC survival and activation by neutrophils and macrophages

In previous studies, both neutrophils and macrophages have been reported to affect HSC functions during liver disease progression^14,29^. However, their influence on HSCs during liver tumorigenesis is largely unknown. Since infiltration of these innate immune cells preceded HSC proliferation and activation in *kras*^*V12*^-induced liver tumorigenesis (Fig. 1), possible roles of neutrophils and macrophages in regulating state of HSCs were investigated. Splice-blocking morpholinos, MO-gcsfr, MO-irf8 and MO-pu.1 were used to inhibit differentiation of neutrophils or macrophages in *kras*^*V12*^-expressing larvae. Effects of these morpholinos were validated by injection in *lyz*+ or *mpeg*+ embryos (Supplementary Fig. S2). As reported by Feng *et al*.^30^, MO-gcsfr completely abrogated neutrophil differentiation but caused no change of the count of macrophages. Similarly as reported by Clay *et al*.^31^, MO-pu.1 repressed macrophage differentiation whereas neutrophil differentiation was unperturbed. As reported by Shiau *et al*.^32^, MO-irf8 favored neutrophil differentiation at the expense of macrophage differentiation, thereby increasing neutrophils and decreasing macrophages. As LPS induce macrophage activation^33^, a combination of MO-gcsfr morpholino injection and LPS treatment inhibited neutrophil differentiation while concurrently stimulating macrophage differentiation (Supplementary Fig. S2).

**Figure 2:**
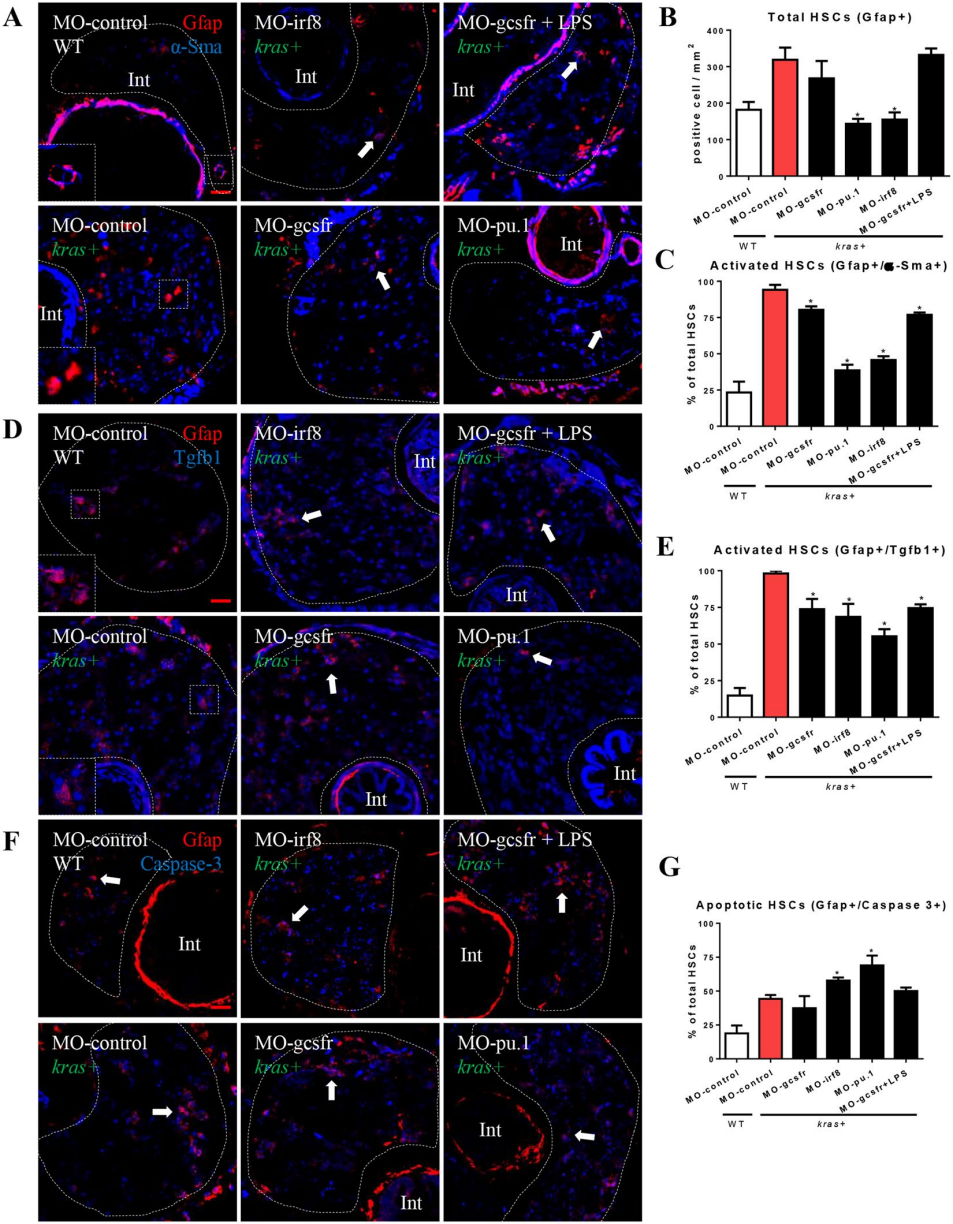
Effects of manipulation of neutrophils and macrophages on HSC density and activation. Wildtype and *kras*+ embryos at one-cell stage were injected with various morpholinos for manipulation of neutrophil and macrophage numbers, and treated with 20 μg/ml dox (or together with LPS) from 3 dpf to 6 dpf. Total HSC number and activated ratio were measured by IF co-staining of Gfap and α-Sma in liver sections. (**A**) IF co-staining of Gfap (red) and a-Sma (blue). (**B**) Quantification of total HSC number (Gfap+ cells). (**C**) Quantification of ratio of activated HSC by counting percentage of α-Sma+ cells in the Gfap+ cell population. (**D**) IF co-staining of Gfap (red) and Tgfb1 (blue). (**E**) Quantification of ratio of Tgfb1+ HSCs. (**F**) IF co-staining of Gfap (red) and Caspase-3 (blue). (**G**) Quantification of ratio of Caspase-3+ apoptotic HSCs. Livers are outlined by dash lines. Arrows indicate examples of co-staining and Int indicates intestine. Insets in (**A**) and (**D**) are enlarged view of representative staining signals in the liver. In all experiments, n = 20 for each group. *P < 0.05, compare to the group in red. Error bars represent biological replicates. Scale bars: 20 μm.

To investigate the effects of neutrophils and macrophages on HSCs, both total and activated HSCs were examined by IF co-staining with Gfap and α-Sma, under different knockdown conditions. Depletion of neutrophils in the *kras*+ larvae by MO-gcsfr or by a combination of MO-gcsfr and LPS treatment did not significantly reduce the numbers of total HSCs but resulted in a small and significant decreases of the portion of activated HSCs (Fig. 2A–C). In contrast, decreasing macrophage counts in the *kras*+ larvae by MO-irf8 and Mo-pu.1 resulted in a decrease in both total and activated HSCs (Fig. 2A–C). As it has been reported that activated HSCs secrete Tgfb1 and promote liver disease progression^34^, to further understand the molecular mechanisms of neutrophil- and macrophage-modulated HSC activity, co-IF staining of Gfap with Tgfb1 was carried out (Fig. 2D,E). As shown in Supplementary Fig. 1B, Tgfb1 is mainly localized in the cytoplasm. Depletion of either neutrophils or macrophages in *kras*+ larvae decreased the percentage of Tgfb1-expressing HSCs, suggesting that both neutrophils and macrophages contribute to Tgfb1-expressing capabilities of HSCs. In contrast, Gfap+/Caspase-3+ cells indicated apoptotic HSCs. Proportion of apoptotic HSCs increased only when macrophage differentiation is inhibited (MO-irf8 and MO-pu.1), thus explaining for the decreased HSC density observed with depletion of macrophages (Fig. 2F,G). Gfap−/Caspase3+ cells are mostly hepatocytes and there was no significant difference of hepatocyte apoptosis between different treatments. Therefore, during *kras*^*V12*^-induced liver tumorigenesis, neutrophils only appear to be required for HSC activation while macrophages contribute to both HSC survival and activation. It is interesting to note from Fig. 2G that *kras* expression, though causing increases of total and activated HSCs, also had a higher apoptotic rate of HSCs; thus it seems that *kras*-induced oncogenesis also leads to a higher turnover of HSCs.

### Serotonin upregulation in hepatocytes, TAMs and Htr2b expression in HSCs

**Figure 3:**
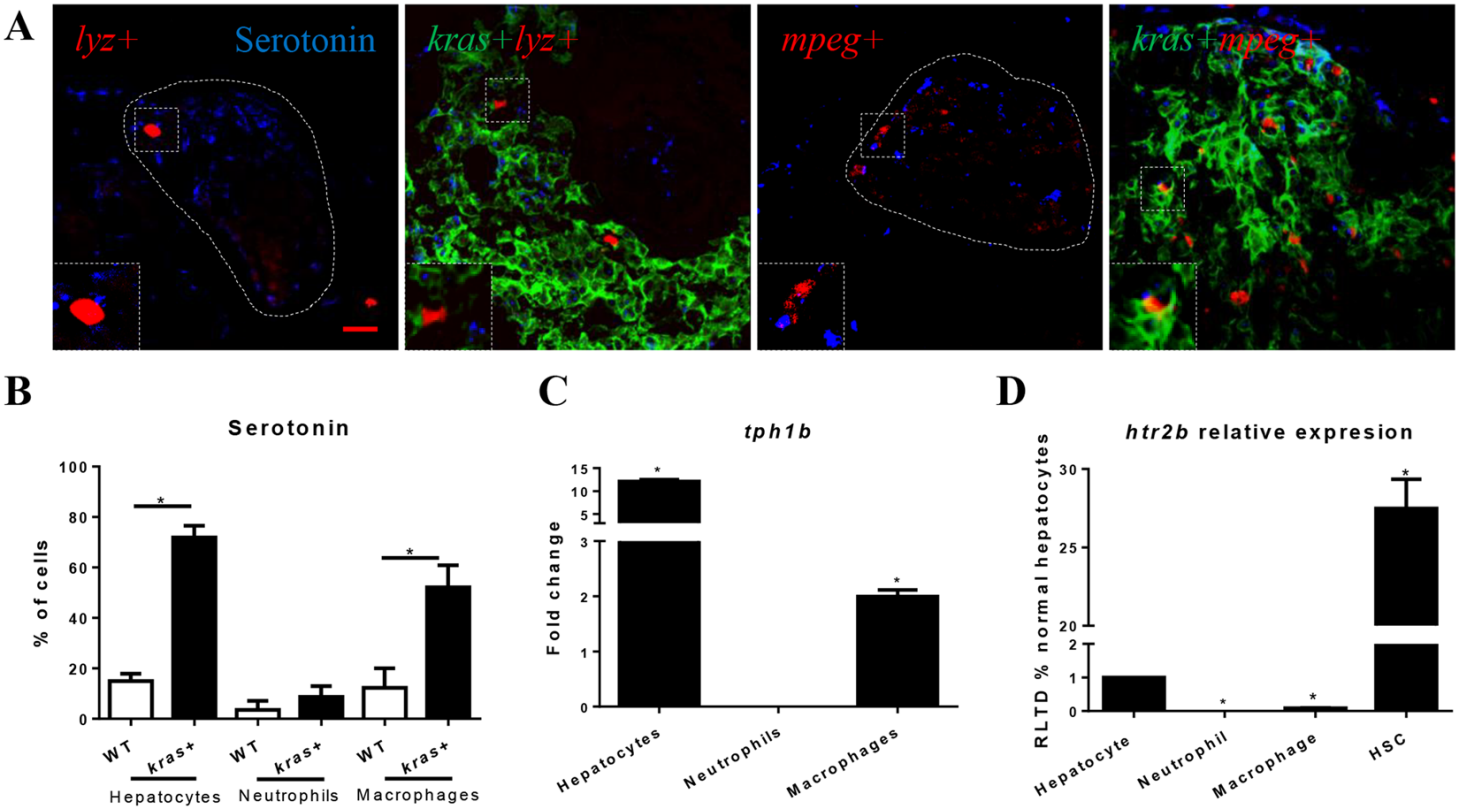
Determination of serotonin in hepatocytes, neutrophils and macrophages. (**A**) Representative images of IF staining of serotonin in liver sections of *kras*+*/lyz*+, *lyz*+ (control), *kras*+*/mpeg*+ and *mpeg*+ (control) larvae (6 dpf). Livers in wildtype larvae are outlined by dash lines. Scale bars: 20 μm. (**B**) Quantification of serotonin+ hepatocytes, neutrophils and macrophages. (**C**) Comparison of *tph1b* expression change after *kras*^*V12*^ induction in hepatocytes, neutrophils and macrophages. (**D**) Predominant expression of *htr2b* mRNA in HSCs. *Htr2b* mRNAs were measured and compared in hepatocytes, neutrophils, macrophages and HSCs with the level expression in hepatocytes was arbitrarily set as 1. Insets in (**A**) are enlarged view of representative staining signals in the liver. In all experiments, n = 20 for each group. *P < 0.05. Error bars represent biological replicates in (**B**) and technical replicates in (**C**) and (**D**). Scale bars: 20 μm.

To explain how TAM maintain HSC survival and activation, possible underlying molecular mechanisms were interrogated. Serotonin served primarily as a neurotransmitter but is also required for HSC survival and activation during liver regeneration^35^. To examine the level of serotonin in hepatocytes, neutrophils and macrophages in wildtype and *kras*^*V12*^-expressing livers, IF staining of serotonin on liver sections of 6 dpf (days post fertilization) *kras*+*/mpeg*+, *mpeg*+, *kras*+*/lyz*+ and *lyz*+ larvae were conducted (Fig. 3A). As shown in Supplementary Fig. 1C, serotonin signal is mainly visiable in the cytoplasm. The serotonin level was significantly upregulated in hepatocytes and TAMs, but not in TANs after *kras*^*V12*^ induction (Fig. 3B). Meanwhile, the expressing of *tph1a*, which encodes the rate-limiting enzyme of serotonin synthesis, tryptophan hydroxylase 1a^36^, was examined and compared in FACS-isolated OHs, TANs and TAMs versus control hepatocytes, naïve neutrophils and macrophages respectively. *Tph1b* expression was found to be significantly up-regulated in OHs and TAMs but not in TANs (Fig. 3C), which was consistent with serotonin upregulation in OHs and TAMs. Serotonin has been showed to play an activating role on HSCs via 5-hydoxytryotamine receptor 2B (Htr2b) in a mouse liver regeneration model^35^. Consistent with this, *htr2b* expression was found to be predominantly in HSCs in zebrafish (Fig. 3D), being about 27 times higher than that in hepatocytes. Almost no *htr2b* expression was detected in neutrophils and macrophages (Fig. 3D).

### Manipulation of Htr2b activity affect liver carcinogenesis and HSC density, activation and function

**Figure 4:**
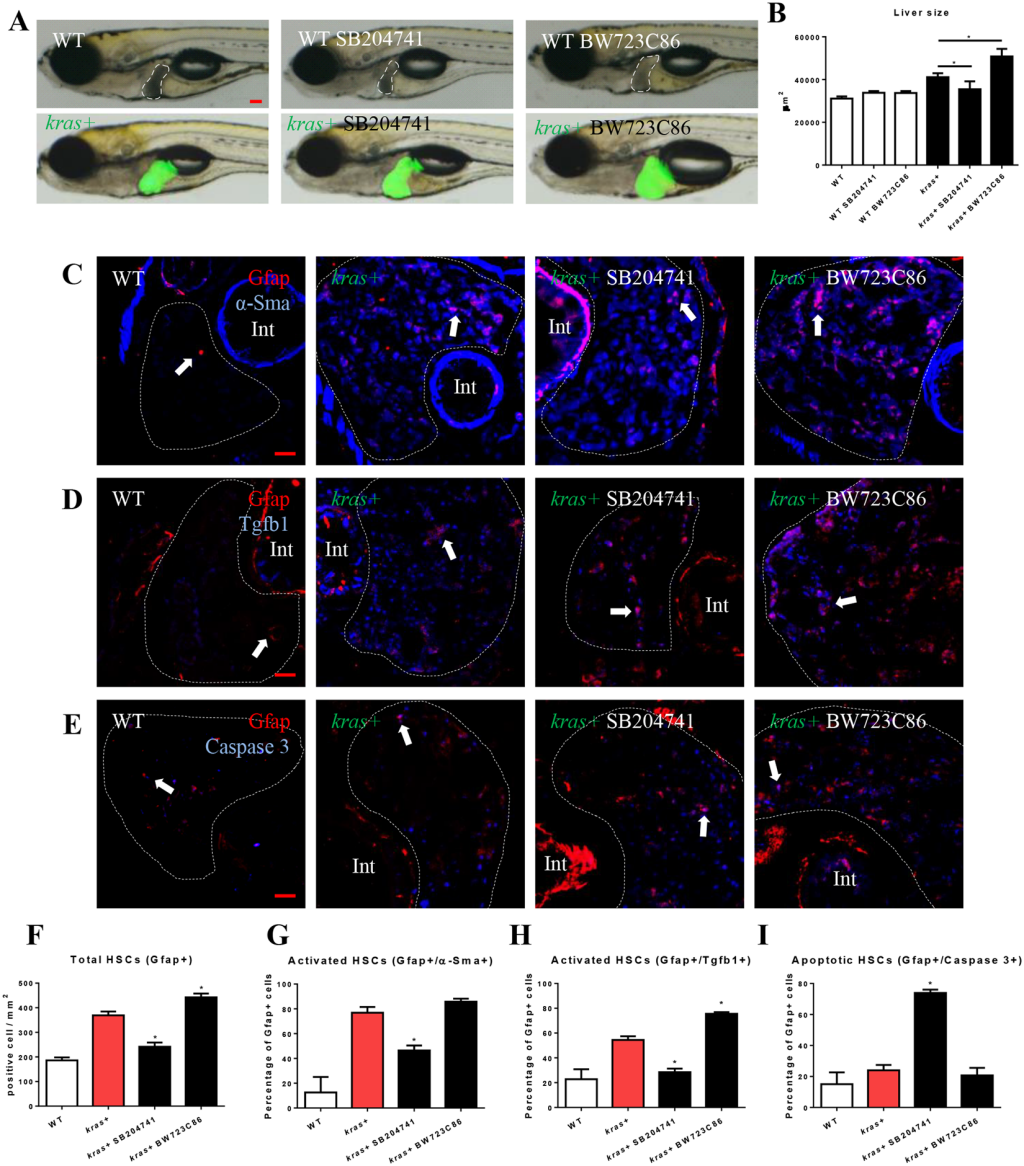
Effects of manipulation of Htr2b on tumorigenesis and HSCs. 3-dpf wildtype or *kras*+ larvae were co-treated with dox and SB204741 or BW723C86 for 4 days. More than 20 fish were analyzed in each group. (**A**) Gross morphology of larvae in left lateral view after 4 days induction. (**B**) Quantification of 2D liver size. (**C**,**F**,**G**) IF co-staining of Gfap and α-Sma on liver sections (**C**) quantification of total HSC density (**F**) and activated HSCs (**G**). (**D**,**H**) IF co-staining of Gfap and Tgfb1 on liver sections (**C**) and quantification of Tgfb1-expressing HSCs (**H**). (**E**,**I**) IF co-staining of Gfap and Caspase-3 on liver sections and quantification of apoptotic HSCs (**I**). Livers are marked by dash lines. Arrows indicate examples of co-staining and Int indicates intestine. In all experiments, n = 20 for each group. *P < 0.05, compare to the the group in red. Error bars represented biological replicates. Scale bar: 20 μm.

To examine the role of Htr2b-activated HSCs in *kras*^*V12*^-induced liver tumorigenesis, *kras*+ larvae was co-treated with doxycycline (dox) and an antagonist (SB204741) or an agonist (BW723C86) of Htr2b. SB204741 has been previously used as an antagonist of Htr2b^35,37^ and it has been shown to have 135 times higher selectivity over the most relevant serotonin receptors, Htr2c^38^. BW723C86 has also been widely used for investigating the effects of activation of Htr2b in disease progression^39–41^. Gross morphology was captured after the treatment and all the larvae were shown in the left lateral view (Fig. 4A). Inhibition of Htr2b in *kras*+ larvae displayed a deterrence of *kras*^*V12*^-induced liver enlargement while stimulation of Htr2b robustly accelerated liver tumorigensis (Fig. 4B). To elucidate the effects on HSC density and activation, IF co-staining of Gfap and α-Sma was performed in the liver section of the larvae fish (Fig. 4C). The antagonist treatment significantly decreased the total HSC density and the ratio of activated HSCs, while the agonist treatment increased HSC density significantly (Fig. 4C,F,G and Supplementary Fig. S3). It has been reported that activated HSCs secrete Tgfb1 to promote liver fibrogenesis^42^. IF co-staining of Gfap and Tgfb1 showed that the antagonist decreased Tgfb1 expression and the agonist promoted Tgfb1 expression (Fig. 4D,H). In addition, IF co-staining of Gfap and Caspase-3 indicated that the antagonist, but not the agonist, significantly induced HSC apoptosis (Fig. 4E,I). In sum, Htr2b was crucial for HSC survival and function; activation of Htr2b induced HSC proliferation and stimulated Tgfb1 expression, while inhibition of Htr2b caused HSC apoptosis.

**Figure 5:**
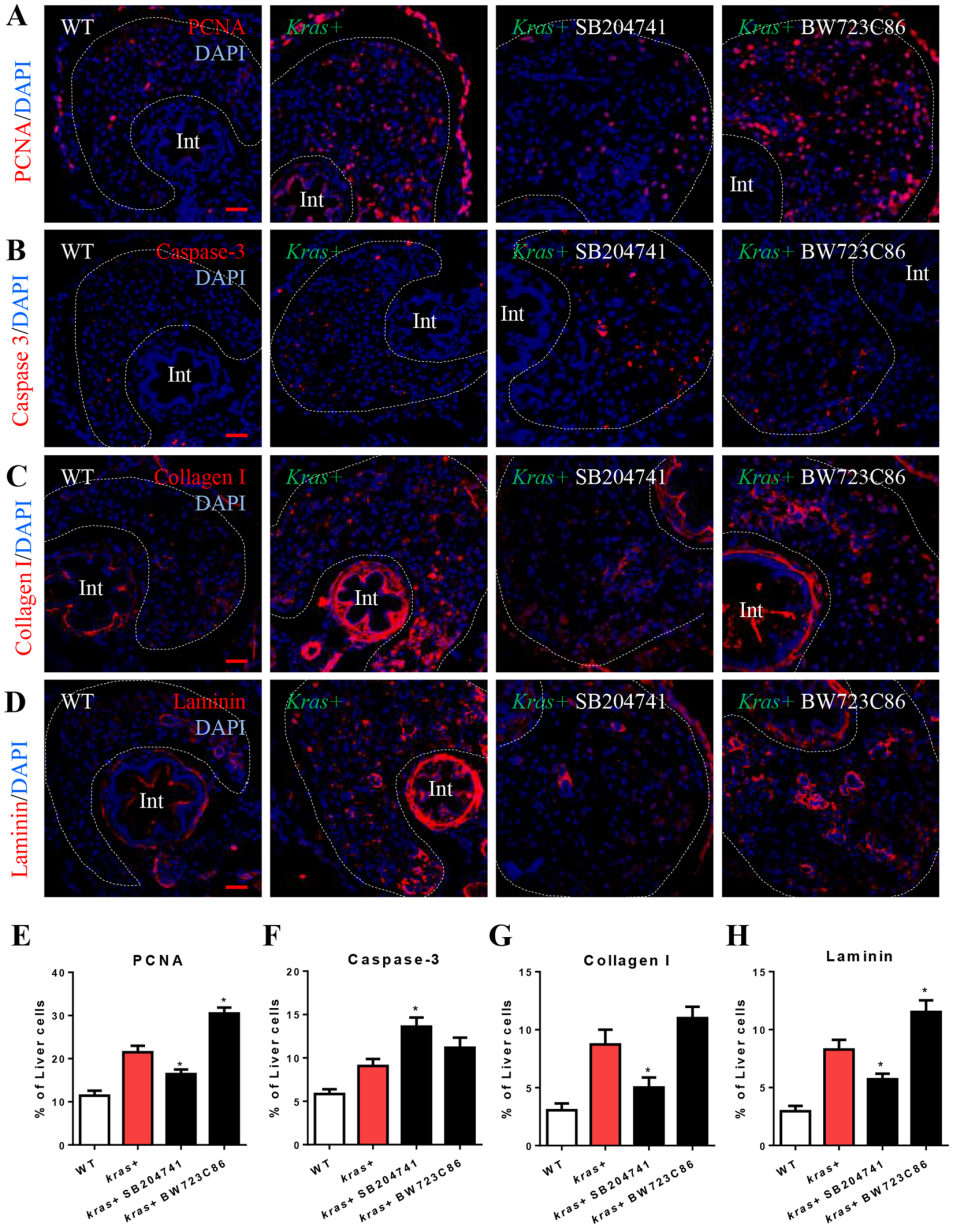
Effects of manipulation of Htr2b on hepatocarcinogenesis and liver fibrosis. 3-dpf wildtype or *kras*+ larvae were co-treated with dox and SB204741 or BW723C86 for 4 days. More than 20 fish were analyzed in each group. (**A**,**E**) IF staining of PCNA on liver sections (**A**) and quantification of PCNA positive cells (**E**). (**B**,**F**) IF staining of Caspase-3 on liver sections (**B**) and quantification of apoptotic cells (**F**). (**C**,**G**) IF staining of collagen I on liver sections (**C**) and quantification of percentage of collagen I positive area (**G**). (**D**,**H**) IF staining of Laminin on liver sections (**D**) and quantification of percentage of Laminin positive area (**H**). Livers are marked by dash lines. Arrows indicate examples of co-staining and Int indicates intestine. In all experiments, n = 20 for each group. *P < 0.05, compare to the the group in red. Error bars represented biological replicates. Scale bar: 20 μm.

To identify the underlying mechanism for the change in liver size caused by inhibition and activation of Htr2b during *kras*^*V12*^-induced carcinogenesis, IF staining of hepatocyte proliferation (PCNA) and apoptosis (Caspase-3) was carried out (Fig. 5A,B and Supplementary Fig. S4A,B). The antagonist treatment significantly inhibited the proliferation of oncogenic hepatocytes and stimulated the hepatocyte apoptosis. The agonist treatment accelerated the hepatocyte proliferation (Fig. 5E,F). However, both antagonist and agonist treatments had no significant effects on cell proliferation and apoptosis in wildtype livers. Furthermore, whether or not the antagonist and agonist treatments affected the progression of liver fibrosis was also investigated. Progression of liver diseases is generally through liver fibrosis to cirrhosis and to carcinoma^43^ and collagen I and Laminin has been widely used as the liver fibrosis markers^44^. To examine the progression level of liver fibrosis, Collagen I and Laminin were detected by IF staining (Fig. 5C,D). The antagonist of Htr2b significantly inhibited the deposition of both Collagen I and Laminin, while the agonist treatment slightly stimulated the progression of liver fibrosis. There were no significant differences in wildtype liver among the two different treatments (Fig. 5G,H). Thus, it seems that the antagonist blocked liver carcinogenesis through induction of cell apoptosis and attenuate liver fibrosis while the agonist stimulated liver carcinogenesis by accelerating hepatocyte proliferation and liver fibrosis.

### Reciprocal roles of HSCs on oncogenic hepatocytes, neutrophils and macrophages

**Figure 6:**
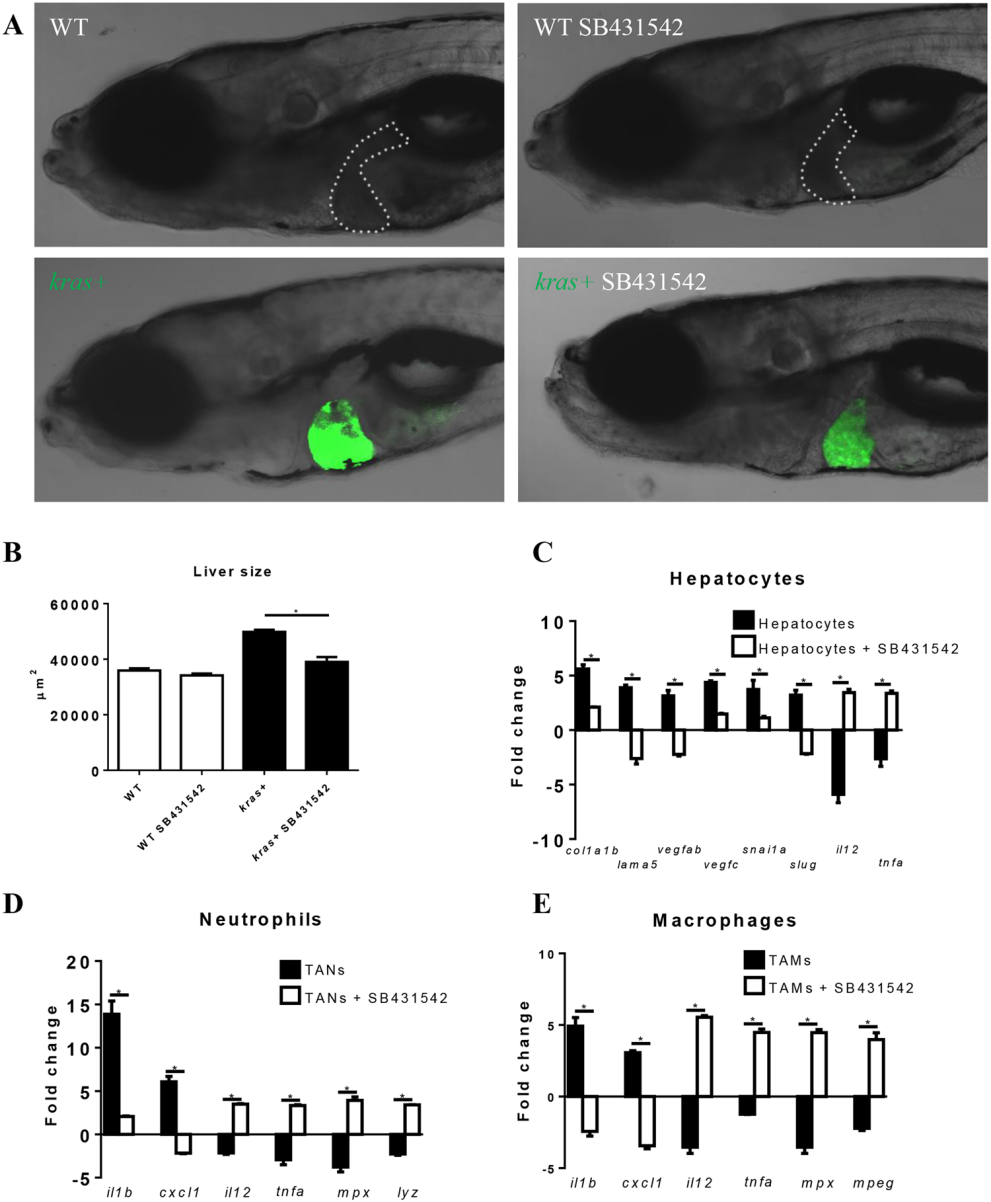
Effect of inhibition of Tgfb signaling on liver tumorigenesis and pro-tumor gene expression in hepatocytes, neutrophils and macrophages. 3-dpf wildtype or *kras*+ larvae were treated with dox and SB431542 for 4 days. (**A**,**B**) Gross morphology of larvae after 4 days treatments (**A**) and quantification of liver size (**B**). (**C–E**) Expression of selected genes for pro-fibrosis, EMT and anti-tumor in hepatocytes (**C**), neutrophils (**D**) and macrophages (**E**). Fold changes refer the expression levels in *kras*+ larvae versus wildtype larvae. In all experiments, n = 20 for each group. *P < 0.05. Error bars represent biological replicates in (**B**) and technical replicates in (**C**), (**D**) and (**E**). Scale bars: 20 μm.

As demonstrated in previous sections (Figs 3 and 4), serotonin was greatly increased upon activation of oncogenic *kras* in hepatocytes and this in turn activated HSCs through the receptor Htr2b. An obvious feature of the activation is the increase of Tgfb1 in activated HSCs, as previously reported for human cystic fibrosis-associated liver disease samples^45^. The serotonin-Tgfb signaling in HSC has been shown in a mouse model where seorotonin binding to Htr2b is found to stimulate *TGFB1* expression in HSCs^35^. In our previous report, we have already shown the up-regulation of Tgfb in HSCs by serotonin treatment in zebrafish^46^. In the present study, the reciprocal role of HSCs on other cell types, such as hepatocytes, neutrophils and macrophages, was also investigated by examining gene expression changes following inhibition of Tgfb signaling. SB431542 is a specific inhibitor of Tgfb type I receptor and has been used in both human cells and zebrafish^47,48^. In our previous study, we also demonstrated the effect of SB431542 in zebrafish larvae by observing a significant decrease of phosphated Smad2, a well recognized downstream effector^25^. Inhibition of Tgfb signaling deterred the oncogene-induced liver enlargement (Fig. 6A,B). To further examine the effect of Tgfb1 on OHs, neutrophils and macrophages, gene expression of FACS isolated hepatocytes, neutrophils and macrophages from *kras*+, *kras*+*/lyz*+ and *kras*+*/mpeg*+ larvae treated with dox or dox/SB431542 was compared to FACS isolated control hepatocytes, naïve neutrophils and macrophages from *fabp10*+, *lyz*+ and *mpeg*+ larvae, respectively. In OHs, *kras*^*V12*^-induced up-regulation of pro-fibrosis (*col1a1b*, *lama5*), angiogenesis (*vegfab*, *vegfc*), epithelial-mesenchyme transition (*snai1*, *slug*) markers and down-regulation of anti-tumor cytokines (*il12*, *tnfa*) were significantly deterred by Tgfb inhibition (Fig. 6C). In TANs and TAMs, *kras*^*V12*^-induced up-regulation of pro-cancer inflammation genes (*il1b* and *cxcl1*) and down-regulation of anti-tumor cytokines (*il12* and *tnfα*) and phagocytosis genes (*mpx*, *lyz* and *mpeg*) were also reverted with Tgfb signaling inhibition (Fig. 6D,E). Together, changes in gene expression profile showed that with Tgfb signaling inhibition, OHs, TANs and TAMs became less pro-cancer inflammation and more anti-tumor.

## Discussion

In clinical studies, microenvironment inflammatory infiltrate has a positive correlation with HCC progression^49^. Liver fibrosis has a positive relevance in a long-term prognosis of nonalcoholic fatty liver disease and HCC^50^. We showed here that there is a rapid modification of microenvironment after oncogene activation. Significant infiltration of neutrophils and macrophages occurred first, followed by activation of HSC at least partially through TANs and/or TAMs. By knocking down the critical genes for neutrophil and macrophage differentiation, we find decreased numbers of neutrophils or macrophages to also cause a significant effect on HSC density and function. Depletion of macrophages decreased both total and activated HSCs, while depletion of neutrophils blocked HSC activation significantly, indicating different crosstalks between HSCs and these innate immune cells in the TME. This observation was reminiscent of previous studies where during liver fibrosis, neutrophils activate HSCs via Il17-dependent mechanism while macrophages is a stimulating factor for HSCs via NFkb activation^29,51^.

Molecularly, serotonin has been reported to activate stellate cells in a liver regeneration context^35^. In clinical studies, serotonin level is significantly increased during liver disease progression and is the highest during cirrhosis and early HCC^52^. Previously, we have found that serotonin is up-regulated in adult zebrafish HCC model and human liver disease samples^46^. Our current study recapitulated the increased serotonin level during *kras*^*V12*^-induced liver tumorigenesis in both OHs and TAMs, but not in TANs in zebrafish larvae. Serotonin are essential for HSC activation and Tgfb1 expression. The serotonin expression in TAMs and OHs, but not in TANs, explained that inhibition of macrophage differentiation had more crucial effects on HSC survival and function than inhibition of neutrophil differentiation. However, depletion of neutrophils also decreased the ratio of activated HSC, implying that there could be other signaling crosstalk between neutrophils and HSCs.

HSCs are well-established source of Tgfb1. In fact, HSC produced Tgfb1 is the pre-eminent mediator of liver fibrosis^45^. Here, we also observed upregulated expression of Tgfb1 after HSC activation. Tgfb1 is a known chemoattractant for both neutrophils^53^ and macrophages^54^ and it has been reported to increase hepatocyte proliferation and polarize neutrophils or macrophages to a pro-tumor profile during hepatocarcinogenesis^55^. After inhibition of Tgfb signaling, our zebrafish model showed downregulation of pro-tumor gene expression profiles in TANs and TAMs, indicating that the activated HSC-produced Tgfb1 could have feedback activation of TANs and TAMs.

In conclusion, with an inducible oncogene model in zebrafish larvae, we elucidated a regulatory mechanism of HSCs via upregulated serotonin in OHs and TAMs. Serotonin activated-HSC produced Tgfb, which in turn activated TANs and TAMs. Hence, microenvironment crosstalk between HSCs and TANs/TAMs could mutually activate one another, thus accelerating liver tumorigenesis. Inhibition of the crosstalk between these cell types could significantly deter rate of carcinogenesis. Hence, targeting these tumor-supporting components offers an attractive therapeutic strategy in the treatment of liver diseases.

## Methods

### Zebrafish husbandry

Zebrafish were maintained in compliance with the guideline and protocol approved by Institutional Animal Care and Use Committee of National University of Singapore. Five transgenic lines, *Tg*(*fabp10:rtTA2s-M2; TRE2:EGFP-krasG12V*) (gz32Tg) in a Tet-On system for inducible, hepatocyte-specific expression of oncogenic *kras*^*G12V*^ ^16^; *Tg*(*lyz:DsRed2*) (nz50Tg) with DsRed-labelled neutrophils under the *lyz* (lysozyme C) promoter^23^; *Tg*(*mpeg1:mCherry*) (gl22Tg), with mCherry-labeled macrophages under the *mpeg1* (macrophage expressed gene 1) promoter^24^; *Tg*(*fabp10:DsRed; ela3l:EGFP*) (gz15Tg) with DsRed-labeled hepatocytes under the *fabp10a* (fatty acid binding protein 10a) promoter and EGFP-labeled exocrine pancreatic cells under the *ela3l* (elastase) promoter^56^ were used and referred to as *kras*+, *lyz*+, *mpeg*+ and *fabp10*+, respectively, in the present report.

### Chemical treatments

All chemical treatments were conducted from 3 dpf for 4 days. The chemicals used included doxycycline (dox) (20 µg/ml; Sigma, D9891), BW723C86 (15 µM; Sigma, B175), SB204741 (15 µM; Sigma, S0693), SB431542 (2.5 µM; Tocris, 1614), LPS (5 ng/µl; Sigma, L4391). The dosages were selected based on our previously validated experiments^25,46,57^.

### Morpholino knock-down

For knockdown of Gcsfr, Irf8 and Pu.1, previously validated gene-specific morpholinos were used, including MO-gcsfr (5′-GAAGCACAAGCGAGACGGATGCCAT-3′)^30^, MO-irf8 (5′-AATGTTTCGCTTACTTTGAAAATGG-3′)^58^ and MO-pu.1 (5′- GATATACTGATACTCCATTGGTGGT-3′)^59^. A standard control morpholino, MO-control (5′-CCTCTTACCTCAGTTACAATTTATA-3′) targeting a human beta-globin intron (Gene Tools, Philomath, OR), was used as a negative control. Aliquots of morpholino (1 mM) and 1% (wt/vol) phenol red in Danieau solution were injected into embryos at the 1-cell stage. Dox was added from 3 dpf to 6 dpf.

### Photography and image analysis

Prior to imaging, zebrafish larvae were anesthetized in tricaine (0.08%; Sigma, E10521) and immobilized in methylcellulose (3%; Sigma, M0521). For each time point of treatment experiments, 20 larvae were used for imaging analyses in each group. Each larva was photographed individually with Olympus microscope. Livers in wildtype fish were manually circled based on bright field images and measured using ImageJ. Tumor livers in *kras*+ fish were imaged for GFP expression and GFP expressing areas were measured by ImageJ.

### Isolation of hepatocytes, HSCs, neutrophils and macrophages by FACS

To enrich HSCs, neutrophils, macrophages and hepatocytes, central part of 6-dpf larvae (after removal of the head and tail regions) were used for FACS using a cell sorter (BD Aria, 643245). The central part of larvae were dissociated into single cells using a 40-µm mesh (BD falcon, 352340) following digestion with trypsin (0.05%; Sigma, T1426), as previously described^60^. *Kras*^*V12*^ expressing hepatocytes (*kras*+) and HSCs (*hand2*+) were isolated based on EGFP expression, neutrophils (*lyz*+) and normal hepatocytes (*fabp*+) were isolated based on DsRed expression and macrophages (*mpeg*+) were isolated based on mCherry expression. Expression of *fabp10a*, *lyz* and *mpeg* was further used to confirm the purity of sorted hepatocytes, neutrophils and macrophages (Supplementary Fig. S5). Indeed, *fabp10a*, *lyz and mpeg* were highly and specifically expressed in hepatocytes, neutrophils and macrophages, respectively in both wildtype and *kras*+ fish.

### RNA extraction, cDNA amplification and RT-qPCR (reverse transcription-quantitative PCR)

Total RNA was extracted using RNeasy mini kit (Qiagen, 74104). A total of 5 ng RNA was used as a template to synthesize and amplify cDNA using QuantiTect Whole Transcriptome Kit (Qiagen, 207043). Amplified cDNA was thus used for real-time quantitative PCR with LightCycler 480 SYBR Green I Master (Roche, 04707516001). Interested genes were amplified for 40 cycles (95 °C, 20 seconds; 65 °C, 15 seconds; 72 °C, 30 seconds). The sequences of primers used are presented in Supplementary Table S1.

### Histological and cytological analyses

Zebrafish larvae were fixed in 4% paraformaldehyde in phosphate buffered saline (Sigma, P6748) and cryo-sectioned at 8 µm thickness using a Leica Cryostats CM1950, followed by immunofluorescence (IF) staining. The primary antibody was derived either from rabbits (anti-PCNA, Santa Cruz, FL-261; anti-Caspase-3, BD biosciences, C92-065; anti-Serotonin, Sigma, C5545; anti-Tgfb1, Anaspec, 55450; anti-Collagen I, Abcam, ab23730; anti-laminin, Sigma, L9393; anti-α-Sma, Abcam, ab15734) or from mice (Anti-Gfap, Abcam, 154474). Anti-mouse or anti-rabbit secondary antibodies were purchased from Thermo Fisher Scientific.

### Statistical analysis

Statistical significance between two groups was evaluated by two-tailed unpaired Student t-test using inStat version 5.0 for Windows (GraphPad, San Diego, CA). Statistical data are presented as mean values ± standard error of mean.

## Electronic supplementary material

Supplementary Figures 1-5 and Supplementary Table 1

## References

[1] Yin C, Evason KJ, Asahina K, Stainier DY (2013). Hepatic stellate cells in liver development, regeneration, and cancer. J Clin Invest.

[2] Lee YA, Wallace MC, Friedman SL (2015). Pathobiology of liver fibrosis: a translational success story. Gut.

[3] Washington K (2000). Hepatic stellate cell activation in nonalcoholic steatohepatitis and fatty liver. Hum Pathol.

[4] Moreira RK (2007). Hepatic stellate cells and liver fibrosis. Arch Pathol Lab Med.

[5] Friedman SL (2008). Hepatic stellate cells: protean, multifunctional, and enigmatic cells of the liver. Physiol Rev.

[6] Llovet JM, Schwartz M, Mazzaferro V (2005). Resection and liver transplantation for hepatocellular carcinoma. Semin Liver Dis.

[7] Sherman M (2008). Recurrence of hepatocellular carcinoma. N Engl J Med.

[8] Yang W (2012). Estrogen represses hepatocellular carcinoma (HCC) growth via inhibiting alternative activation of tumor-associated macrophages (TAMs). J Biol Chem.

[9] Li YW (2011). Intratumoral neutrophils: a poor prognostic factor for hepatocellular carcinoma following resection. J Hepatol.

[10] Ju MJ (2009). Peritumoral activated hepatic stellate cells predict poor clinical outcome in hepatocellular carcinoma after curative resection. Am J Clin Pathol.

[11] Hernandez-Gea V, Toffanin S, Friedman SL, Llovet JM (2013). Role of the microenvironment in the pathogenesis and treatment of hepatocellular carcinoma. Gastroenterology.

[12] Wu K, Kryczek I, Chen L, Zou W, Welling TH (2009). Kupffer cell suppression of CD8+ T cells in human hepatocellular carcinoma is mediated by B7-H1/programmed death-1 interactions. Cancer Res.

[13] Pradere JP (2013). Hepatic macrophages but not dendritic cells contribute to liver fibrosis by promoting the survival of activated hepatic stellate cells in mice. Hepatology.

[14] Casini A (1997). Neutrophil-derived superoxide anion induces lipid peroxidation and stimulates collagen synthesis in human hepatic stellate cells: role of nitric oxide. Hepatology.

[15] White R, Rose K, Zon L (2013). Zebrafish cancer: the state of the art and the path forward. Nat Rev Cancer.

[16] Chew TW (2014). Crosstalk of Ras and Rho: activation of RhoA abates Kras-induced liver tumorigenesis in transgenic zebrafish models. Oncogene.

[17] Li Z (2012). Inducible and repressable oncogene-addicted hepatocellular carcinoma in Tet-on xmrk transgenic zebrafish. J Hepatol.

[18] Li Z (2013). A transgenic zebrafish liver tumor model with inducible Myc expression reveals conserved Myc signatures with mammalian liver tumors. Disease models & mechanisms.

[19] Nguyen AT (2012). An inducible kras(V12) transgenic zebrafish model for liver tumorigenesis and chemical drug screening. Disease models & mechanisms.

[20] Zheng W (2014). Xmrk, kras and myc transgenic zebrafish liver cancer models share molecular signatures with subsets of human hepatocellular carcinoma. PLoS One.

[21] Lam SH (2006). Conservation of gene expression signatures between zebrafish and human liver tumors and tumor progression. Nat Biotechnol.

[22] Yin C, Evason KJ, Maher JJ, Stainier DY (2012). The basic helix-loop-helix transcription factor, heart and neural crest derivatives expressed transcript 2, marks hepatic stellate cells in zebrafish: analysis of stellate cell entry into the developing liver. Hepatology.

[23] Hall C, Flores MV, Storm T, Crosier K, Crosier P (2007). The zebrafish lysozyme C promoter drives myeloid-specific expression in transgenic fish. BMC Dev Biol.

[24] Ellett F, Pase L, Hayman JW, Andrianopoulos A, Lieschke GJ (2011). mpeg1 promoter transgenes direct macrophage-lineage expression in zebrafish. Blood.

[25] Yan C, Huo X, Wang S, Feng Y, Gong Z (2015). Stimulation of hepatocarcinogenesis by neutrophils upon induction of oncogenic kras expression in transgenic zebrafish. J Hepatol.

[26] Carpino G (2005). Alpha-SMA expression in hepatic stellate cells and quantitative analysis of hepatic fibrosis in cirrhosis and in recurrent chronic hepatitis after liver transplantation. Dig Liver Dis.

[27] Talele NP, Fradette J, Davies JE, Kapus A, Hinz B (2015). Expression of alpha-Smooth Muscle Actin Determines the Fate of Mesenchymal Stromal Cells. Stem Cell Rep.

[28] Chen, J. *et al*. Vitamin D Deficiency Promotes Liver Tumor Growth in Transforming Growth Factor-beta/Smad3-Deficient Mice Through Wnt and Toll-likeReceptor 7 Pathway Modulation. S*ci Rep-Uk* 6, doi:ARTN 3021710.1038/srep30217 (2016). 10.1038/srep30217 PMC4960540 27456065

[29] Imamura M, Ogawa T, Sasaguri Y, Chayama K, Ueno H (2005). Suppression of macrophage infiltration inhibits activation of hepatic stellate cells and liver fibrogenesis in rats. Gastroenterology.

[30] Feng Y, Renshaw S, Martin P (2012). Live imaging of tumor initiation in zebrafish larvae reveals a trophic role for leukocyte-derived PGE(2). Curr Biol.

[31] Clay H (2007). Dichotomous role of the macrophage in early Mycobacterium marinum infection of the zebrafish. Cell Host Microbe.

[32] Shiau CE, Kaufman Z, Meireles AM, Talbot WS (2015). Differential requirement for irf8 in formation of embryonic and adult macrophages in zebrafish. PLoS One.

[33] Meng F, Lowell CA (1997). Lipopolysaccharide (LPS)-induced macrophage activation and signal transduction in the absence of Src-family kinases Hck, Fgr, and Lyn. J Exp Med.

[34] Giannelli G, Villa E, Lahn M (2014). Transforming growth factor-beta as a therapeutic target in hepatocellular carcinoma. Cancer Res.

[35] Ebrahimkhani MR (2011). Stimulating healthy tissue regeneration by targeting the 5-HT(2)B receptor in chronic liver disease. Nat Med.

[36] Heredia DJ (2013). Important role of mucosal serotonin in colonic propulsion and peristaltic reflexes: *in vitro* analyses in mice lacking tryptophan hydroxylase 1. J Physiol.

[37] West JD (2016). Serotonin 2B Receptor Antagonism Prevents Heritable Pulmonary Arterial Hypertension. PLoS One.

[38] Forbes IT, Jones GE, Murphy OE, Holland V, Baxter GS (1995). N-(1-methyl-5-indolyl)-N’-(3-methyl-5-isothiazolyl)urea: a novel, high-affinity 5-HT2B receptor antagonist. J Med Chem.

[39] Oh EJ (2016). A Novel Role of Serotonin Receptor 2B Agonist as an Anti-Melanogenesis Agent. Int J Mol Sci.

[40] Gunther S, Maroteaux L, Schwarzacher SW (2006). Endogenous 5-HT2B receptor activation regulates neonatal respiratory activity *in vitro*. J Neurobiol.

[41] Cataldo LR (2017). Prolonged Activation of the Htr2b Serotonin Receptor Impairs Glucose Stimulated Insulin Secretion and Mitochondrial Function in MIN6 Cells. PLoS One.

[42] Tahashi Y (2002). Differential regulation of TGF-beta signal in hepatic stellate cells between acute and chronic rat liver injury. Hepatology.

[43] El-Mezayen HA, Habib S, Marzok HF, Saad MH (2015). Diagnostic performance of collagen IV and laminin for the prediction of fibrosis and cirrhosis in chronic hepatitis C patients: a multicenter study. Eur J Gastroenterol Hepatol.

[44] Mak KM, Chen LL, Lee TF (2013). Codistribution of collagen type IV and laminin in liver fibrosis of elderly cadavers: immunohistochemical marker of perisinusoidal basement membrane formation. Anat Rec (Hoboken).

[45] Lewindon PJ (2002). The role of hepatic stellate cells and transforming growth factor-beta(1) in cystic fibrosis liver disease. Am J Pathol.

[46] Yang Q, Yan C, Yin C, Gong Z (2017). Serotonin Activated Hepatic Stellate Cells Contribute to Sex Disparity in Hepatocellular Carcinoma. Cell Mol Gastroenterol Hepatol.

[47] Sun Z (2006). Activation and roles of ALK4/ALK7-mediated maternal TGFbeta signals in zebrafish embryo. Biochemical and biophysical research communications.

[48] Halder SK, Beauchamp RD, Datta PK (2005). A Specific Inhibitor of TGF-β Receptor Kinase, SB-431542, as a Potent Antitumor Agent for Human Cancers. Neoplasia.

[49] Critelli R (2017). Microenvironment inflammatory infiltrate drives growth speed and outcome of hepatocellular carcinoma: a prospective clinical study. Cell Death Dis.

[50] Angulo P (2015). Liver Fibrosis, but No Other Histologic Features, Is Associated With Long-term Outcomes of Patients With Nonalcoholic Fatty Liver Disease. Gastroenterology.

[51] Tan Z (2013). IL-17A plays a critical role in the pathogenesis of liver fibrosis through hepatic stellate cell activation. J Immunol.

[52] Abdel-Razik A (2016). Could serotonin be a potential marker for hepatocellular carcinoma? A prospective single-center observational study. Eur J Gastroenterol Hepatol.

[53] Reibman J (1991). Transforming growth factor beta 1, a potent chemoattractant for human neutrophils, bypasses classic signal-transduction pathways. Proc Natl Acad Sci USA.

[54] Kim JS (2006). Transforming growth factor-beta1 regulates macrophage migration via RhoA. Blood.

[55] Cortez-Retamozo V (2012). Origins of tumor-associated macrophages and neutrophils. Proc Natl Acad Sci USA.

[56] Korzh S (2008). Requirement of vasculogenesis and blood circulation in late stages of liver growth in zebrafish. BMC Dev Biol.

[57] Yan C, Yang Q, Gong Z (2017). Tumor-Associated Neutrophils and Macrophages Promote Gender Disparity in Hepatocellular Carcinoma in Zebrafish. Cancer Res.

[58] Li L, Jin H, Xu J, Shi Y, Wen Z (2011). Irf8 regulates macrophage versus neutrophil fate during zebrafish primitive myelopoiesis. Blood.

[59] Bukrinsky A, Griffin KJ, Zhao Y, Lin S, Banerjee U (2009). Essential role of spi-1-like (spi-1l) in zebrafish myeloid cell differentiation. Blood.

[60] Manoli, M. & Driever, W. Fluorescence-activated cell sorting (FACS) of fluorescently tagged cells from zebrafish larvae for RNA isolation. *Cold Spring Harb Protoc* **2012**, 10.1101/pdb.prot069633 (2012). 10.1101/pdb.prot069633 22854565

