# Supplementary material for: Interaction of hepatic stellate cells with neutrophils and macrophages in the liver following oncogenic kras activation in transgenic zebrafish
Source: Sci Rep. 2018 May 31;8:8495. doi: 10.1038/s41598-018-26612-0 (PMC5981472; doi:10.1038/s41598-018-26612-0)
Supplement: Supplementary file 1 — Supplementary Figures 1-5 and Supplementary Table 1 [file 41598_2018_26612_MOESM1_ESM.pdf]

Supplementary Information

Interaction of hepatic stellate cells with neutrophils and macrophages in the liver following oncogenic *kras* activation in transgenic zebrafish

Qiqi Yang[1, †], Chuan Yan[1,2, †], Zhiyuan Gong[1,2,*]

[†] These authors contributed equally.

[1]Department of Biological Sciences, [2]National University of Singapore graduate school for integrative sciences and engineering, National University of Singapore, Singapore

[*]Corresponding author, Dr. Zhiyuan Gong, Department of biological Sciences, National University of Singapore, dbsgzy@nus.edu.sg; phone: (65)2-65162860; fax: (65)-67792486.14 Science Drive 4, Singapore

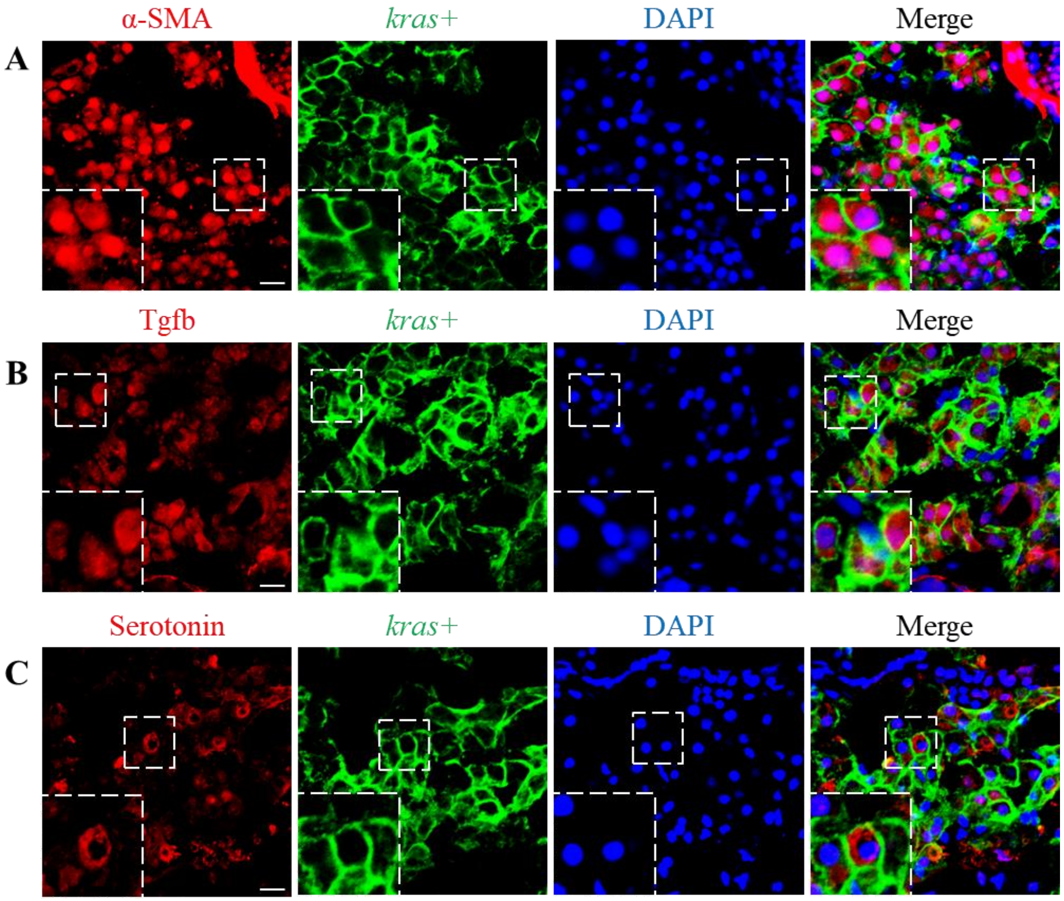

Supplementary Figure S1. Cellular localization of α-SMA, Tgfb and Serotonin. 3-dpf *kras*+ larvae were treated with dox for 4 days and IF staining was carried out for α-SMA (A) Tgfb (B) and serotonin (C). Representative images of liver sections are shown. GFP signals were derived from GFP-Kras fusion protein in *kras*+ fish and are mainly in hepatocyte membrane and cortex region as we previously reported (reference 16). DAPI was count-stained for visualization of nuclei. Boxed regions are enlarged as insets in the left-bottom corners. Scale bar, 10 μm.

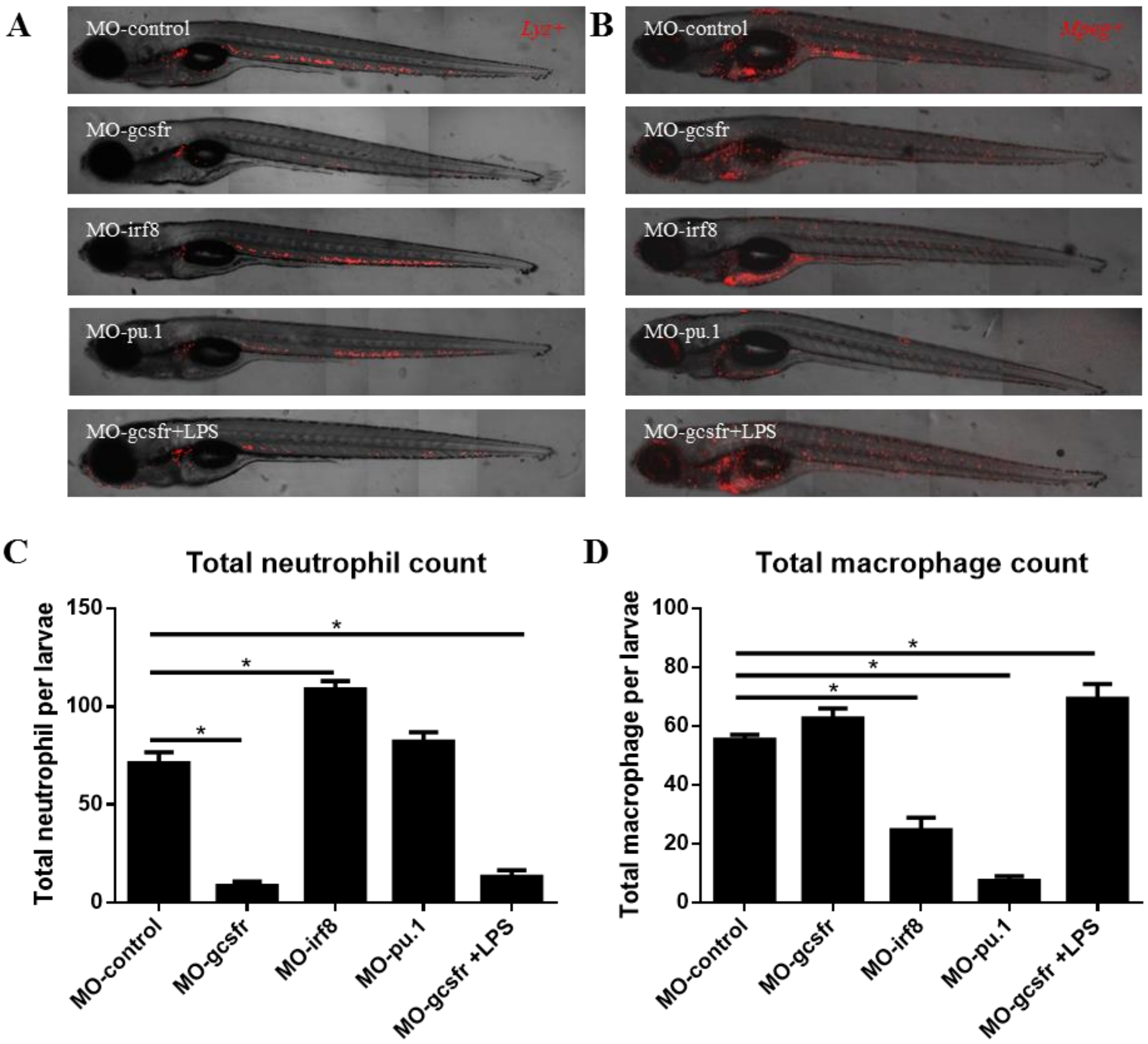

Supplementary Figure S2. Effects of morpholinos on inhibition of differentiation of neutrophils or macrophages. *lyz+* or *mpeg+* fish were crossed with wildtype and morpholinos were injected into the yolk at one cell stage. (A) Representative images of *lyz+* larvae after 6 days of morpholino injection. (B) Representative images of *mpeg+* larvae after 6 days of morpholino injection. (C) Quantification of total neutrophil number in *lyz+* larvae after 6 days of morpholino injection. (D) Quantification of total macrophage number in *mpeg+* larvae after 6 days of morpholino injection. *$P < 0.05$.

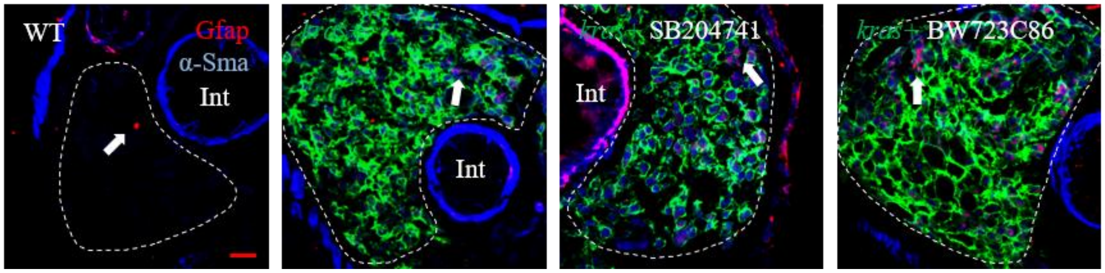

Supplementary Figure S3. Merged image of GFAP, α-Sma and *kras*-EGFP to supplement Fig. 4C.

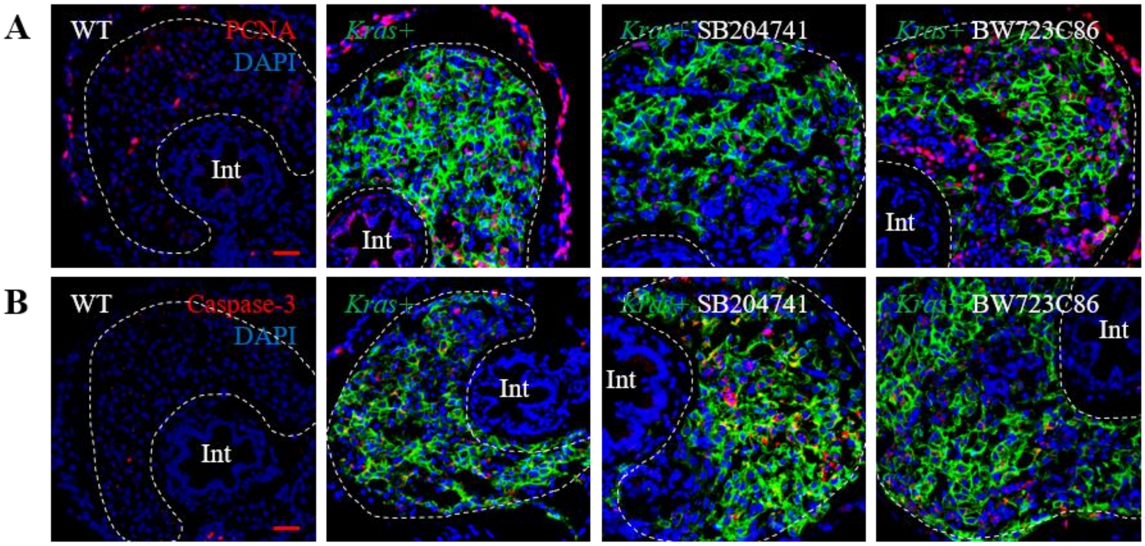

Supplementary Figure S4. (A) Merged images of PCNA, DAPI and *kras*-EGFP to supplement Fig. 5A. (B) Merged images of Caspase 3, DAPI and *kras*-EGFP to supplement Fig. 5B.

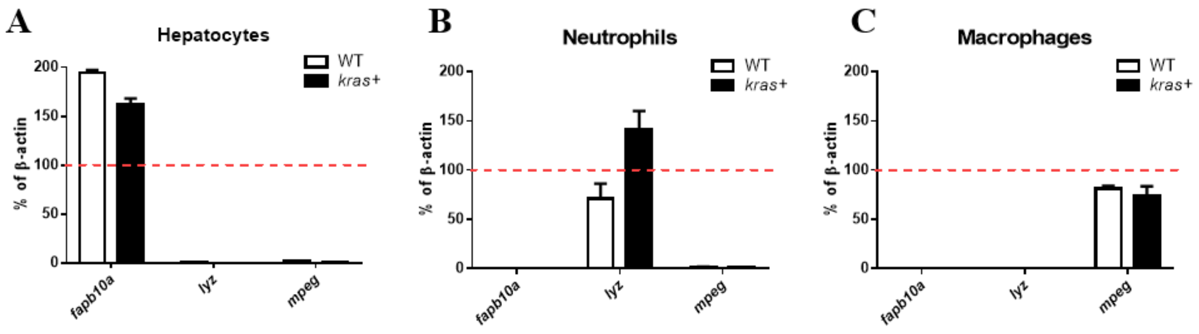

Supplementary Figure S5. RT-qPCR analyses of RNA expression of *fabp10a*, *lyz* and *mpeg* in FACS-isolated hepatocytes (A), neutrophils (B) and macrophages (C). Relative expression levels are shown as percentages of internal β-actin expression level.

Table S1. Sequence of real-time primers

Gene	Forward	Reverse
β-actin	CTCTGGGTCACCGCTTCTTT	CAGATGCTCACGAAACCCCT
tph1b	CTAAGAGCATACGGGGCTGG	GGACGCTGGATTGTCTTTGC
htr2b	CGCAGGATGTCCACCATAGG	TGCACCTTTCACAGAGCACA
col1a1b	TGGCATGACCGGCCCTATTG	CTCTCCTTTAGCACCAGGCT
lama5	GGACCCAAATGCAAGCAACC	CAGAACCCGAGGCTGTATAG
vegfab	TGTTCGCGTGCTCCAGTTAT	GAGGACGGCAGAGAGACTTG
vegfc	TGTGAATGCACAGAGTCCCC	TTCGCACTGCTTCAAATGCC
snai1a	CGGGCACAATATTCAACAGG	GTCAGGGCACCTGTTAGCAC
slug	AAACTGACCCAAGACAGCCT	AGTACGCATCAGCTCAACCC
il12	AGGGCTCTTCGTTTGACGAC	TGTCATGCGGTGGTGTAGTG
tnfa	GCGCTTTTCTGAATCCTACG	TGCCCAGTCTGTCTCCTTCT
il1b	GCCTGTGTGTTTGGGAATCT	TGATAAACCAACCGGGACAT
cxcl1	GGCATTCACACCCAAAGCG	GCGAGCACGATTCAGGAGAG
mpx	TTCTTTTTGTGGTGGGCTGC	AGTCTCAGCGTTTGTGCCAT
lyz	TGTGTCTGGCGTGGATGTCC	TGTTTGCGCTGCTCACAGC
mpeg	CCCACCAAGTGAAAGAGG	GTGTTTGATTGTTTTCAATGG
